# ‘It depends’: Characterizing speech and language therapy for preschool children with developmental speech and language disorders

**DOI:** 10.1111/1460-6984.12498

**Published:** 2019-09-17

**Authors:** Lydia Morgan, Julie Marshall, Sam Harding, Gaye Powell, Yvonne Wren, Jane Coad, Sue Roulstone

**Affiliations:** ^1^ Bristol Speech and Language Therapy Research Unit North Bristol NHS Trust Bristol UK; ^2^ Health Professions Department, Faculty of Health, Psychology and Social Care Manchester Metropolitan University Manchester UK; ^3^ Independent Consultant Torpoint UK; ^4^ University of Bristol Bristol UK; ^5^ University of Nottingham Nottingham UK; ^6^ University of the West of England Bristol UK

**Keywords:** eclectic intervention, model of therapy, preschool children, developmental speech and language disorder

## Abstract

**Background:**

Several studies have suggested that practitioners hold speech and language therapy (SLT) practice as tacit and consequently it is difficult for the therapist to describe. The current study uses a range of knowledge elicitation (KE) approaches, a technique not used before in SLT, as a way of accessing this tacit knowledge. There is currently no agreed framework that establishes key factors underpinning practice for preschool children with speech and language disorders. This paper attempts to address that gap.

**Aims:**

To develop a framework of SLTs’ practice when working with preschool children with developmental speech and language disorders (DS&LD).

**Methods & Procedures:**

A mixed‐methods approach was adopted for this study. Data were collected iteratively, from 245 SLTs with experience of working with preschool children with DS&LD across sites in England, by means of focus groups and national events. There were three stages of data collection: local sites, specific‐interest groups and two national events. KE techniques were used to gather data, with initial data being collected in local site focus groups. Findings from groups were taken to subsequent larger groups where a combination of concept mapping, teach‐back and sorting exercises generated a more detailed description of practice, using discussion of consensus and disagreement to stimulate further exploration and definition and provide validatory evidence.

**Outcomes & Results:**

This paper provides a high‐level framework of therapy for preschool children with DS&LD that makes practice explicit in this area. The framework proposes that therapists’ aims for this group of children fall into three categories: addressing children's areas of impairment and skills; achieving functionally meaningful skills and carryover; and supporting adults to provide a supportive communication environment. The exact configuration is shaped by the child's context and needs.

**Conclusions & Implications:**

The framework highlights themes that are well researched in the literature (e.g., speech) and others that have been little studied (e.g., adult understanding), indicating a disconnect between research evidence and practice. The research also highlights the complex nature of interventions for preschool children with DS&LD and the importance therapists attribute to tailoring therapy to individual needs. The framework provides a scaffold upon which SLTs can focus their clinical practice and encourages the profession to understand and explore better the gaps between research evidence and clinical practice for preschool children with DS&LD.

**What this paper adds**

**What is already known on the subject**

Although there are some existing models of practice, there is currently no agreed conceptualization or consensus of practice for preschool children with DS&LD.

**What this paper adds to existing knowledge**

This paper provides a high‐level conceptual framework of therapy for preschool children with DS&LD. KE techniques were used for the first time with SLTs to provide evidence to design a framework and make explicit the characteristics of practice for preschool children with DS&LD. The study highlights how therapists individualize therapy for this population.

**What are the potential or actual clinical implications of this work?**

The framework encourages therapists to reflect on their practice and provides a structure to help bridge the gap between research evidence and clinical practice and it can be used in the future training of SLTs.

## Introduction

Evidence‐based practice (EBP) is an accepted principle of best practice throughout the speech and language therapy (SLT) profession, as demonstrated by the support of professional bodies. For example, the Royal College of Speech and Language Therapists (RCSLT) provides a clinical decision‐support tool that works through the process of evidence‐based decisions (RCSLT 2018). Similarly, therapists at the University of Sydney developed SpeechBITE (2018), a searchable database focusing on interventions, graded according to study design; while the American Speech–Language–Hearing Association (ASHA) has a ‘compendium’ of evidence‐based guidelines and systematic reviews (ASHA 2018). Nonetheless, surveys indicate that the implementation of EBP has been challenging, with therapists relying on clinical experience rather than research evidence to make their decisions (O'Connor and Pettigrew 2009, Zipoli and Kennedy 2005).

A wide‐ranging review of EBP concluded that the uptake of research by the SLT profession continues to be problematic because of both the nature of research and its use within the profession (McCurtin and Roddam 2012). They point to difficulties in the utility and relevance of research and reliance on randomized controlled trials, which do not replicate practice. They identify the influence of the working context of therapists, including constraints of time and departmental cultures which can work against the routine use of research evidence (McCurtin and Roddam 2012).

In their seminal publication, Argyris and Schön (1974) suggest that practitioners evolve their own ‘theories of practice’. These theories relate to the contexts in which practitioners work and help them to organize their knowledge in ways that are maximally useful in practice (Boschuizen and Schmidt 2000). This is supported by a survey of SLT practice (Law *et al*. 2008), which found that therapists develop their own theories of therapy. This can create challenges to implementing research in practice; while in research, interventions are provided in well‐controlled conditions, in practice SLTs frame their interventions using their theories of practice, adapting interventions to the heterogeneity of children on their caseload, as well as responding to the local context. Understanding the models, explicit or implicit, that therapists use in their daily practice could enable researchers to design studies that reflect practitioners’ theories of practice and to present their findings in a way that maximizes the chance of integration into practice.

To date, attempts to support the implementation of EBP have focused on enhancing practitioners’ ability to access research and to search and critically appraise the evidence. Little research examines EBP from the perspective of the therapists, that is, looking at their everyday practice. The studies that have tried to understand practitioner perspectives on practice in children's services (e.g., Law *et al*. 2008, Roulstone *et al*. 2012a) conclude that much of practitioners’ knowledge is tacit, and only becomes explicit under situations where they reflect on their practice.

A striking feature of SLT practice is the variation in how it is implemented. Through interviews and a survey of practice, Roulstone *et al*. (2012a) identified over 150 interventions used with children and young people with speech, language and communication needs. They found that there was variation in the way interventions were described, including the names of published programmes, types of activities and resources employed, and principles or approaches. Elsewhere, interventions for more specific populations have been explored, for example, for school‐age children with receptive language impairment (Law *et al*. 2008); even in this relatively homogeneous group, wide differentiations in therapies described were identified.

The variation in practice creates an additional barrier to the implementation of EBP, as it is difficult to generate guidance that is applicable across the range of interventions delivered. In order to navigate variations in the context of EBP, it is helpful to have a framework that makes explicit the key tenets of therapy in practice. Evidence‐based approaches can then be mapped against practice and an acceptable range of variation within practice considered. Studies that have explored practice from the perspective of practitioners (Law *et al*. 2008, Roulstone *et al*. 2012a) have not examined therapy with preschool populations. Further, there are no known attempts to explicitly provide a framework of therapy from the perspective of SLTs who work with these populations. This paper addresses this gap.

### Aim

The aim of this study was to develop a framework of SLT practice when working with preschool children with developmental speech and language disorders (DS&LD). Specifically, the objective was to establish the range of opinion, disagreement and consensus around key principles and components of SLT practice with this population. By making current practice explicit, the research aimed to bridge the gap between practice, theories of therapy and evidence of interventions.

## Methods and procedures

### Design

An exploratory mixed‐methods approach was used; data were collected using both quantitative and qualitative methods. The study drew on data‐collection techniques used in the field of artificial intelligence to elicit the knowledge of experts for the purpose of designing decision support software. In this context, the process of asking experts to describe their practice is known as knowledge elicitation (KE) (Shadbolt and Smart 2015). KE techniques are used to capture the conceptual and procedural knowledge of professions or organizations. KE techniques used in this study included focus groups (FGs), concept mapping, sorting and teach‐back processes (Johnson and Johnson 1987, Crandall *et al*. 2006), designed to stimulate reflection on the more tacit aspects of practitioner knowledge. Brief information is provided below of the data‐collection process. Further information about the particular techniques and processes used is provided in the appendix.

The methodology of the study received a favourable opinion by the NHS Research Ethics Committee (11/SW/0228).

### Participants

Perspectives of SLTs, with expertise working with preschool (aged 2;00–5;11) children with DS&LD, were gathered. Inclusion criteria for initial data collection required participants to be currently working in England, to have two or more years of experience as a qualified SLT and to be working with children with DS&LD. For the national events a more flexible criterion was used, participants were required to be qualified SLTs who had an interest in and had worked with preschool children with DS&LD.

### Sampling and recruitment

Three stages of data collection moved from local group, to regional and then national levels; these are described in turn below. Table 1 provides a summary of the participants, data collection, and analysis for each of the three rounds of data collection.

- Round 1: Six local sites. In the first stage, six National Health Service (NHS) SLT services in England were recruited purposively to include services providing for a range of populations, for example, urban and rural, different socioeconomic groups. At each site, the SLT service lead emailed therapists who worked with preschool children with DS&LD, inviting them to participate in the study.
- Round 2: Four special‐interest groups (SIGs). SLT preschool/early years SIGs were identified through adverts in SLT magazines and social networks. Seven SIGs (now called clinical excellence networks) were invited to participate and four subsequently agreed to host a research event.
- Round 3: Two national events were held in Leeds and London. These events were advertised nationally.

**Table 1:** Participants, activities and analysis for each round

	Participants	Average years since qualifying	Activities undertaken	Analysis
*Round 1: Focus groups*				
Site 1	8	5	Focus groups	Content, thematic analysis
Site 2	8	15		
Site 3	8	17		
Site 4	7	12		
Site 5	4	20		
Site 6	5	17		
				
*Round 2: Special‐interest groups*				
SIG 1	16	10	Sorting, concept mapping, teach‐back, validation tasks	Descriptive statistics, framework, thematic analysis
SIG 2	13	14		
SIG 3	18	11		
SIG 4	19	13		
				
*Round 3: National events*				
Leeds	42	–	Sorting, validation tasks	Descriptive statistics
London	44	–		

Notes: Sorting tasks = does the model cover everything that therapists do with children with DS&LD; is each theme essential, desirable or not used?

Concept mapping = how do the themes fit together?

Teach‐back = participants describe how they would explain each theme to a parent.

### Data collection

The research aimed to generate a progressively more detailed description of practice, using discussion of consensus and disagreement to stimulate further exploration and definition. A summary of the data collection and analysis is provided in Table 2.

- Focus groups (FGs) (Round 1): nine FGs were held across six sites. These were semi‐structured, following a topic guide (Roulstone *et al*. 2015: 271) that started with an open question; participants were asked to describe the interventions they use with preschool children with DS&LD. Prompts encouraged participants to provide detail, avoid use of intervention brand names and to provide rationale for interventions. The analysis revealed a number of themes that described the purposes of therapy; these were the focus of the subsequent data collection. An abbreviated topic guide is provided in the appendix.
- Concept mapping (Round 2, SIGs): participants were asked to consider the themes identified in the FGs above to develop their own models of how themes fit together. Participants were given the choice to either draw models or describe them textually. They worked individually or in small groups, with most opting to complete the task with others. Participants were asked to explain their models to the group; field notes recorded their comments.
- Teach‐back (Round 2, SIGs): teach‐back is a process whereby understanding of a procedure is checked by teaching the procedure to someone else or back to the person who demonstrated the procedure (Crandall *et al*. 2006, Shadbolt and Smart 2015). Using this technique, participants provided descriptions of themes identified in the FGs, as if they were describing them to primary caregivers in their service. This allowed checking of theme descriptions against those that had been generated from the initial data set.
- Sorting tasks (Rounds 2 and 3, SIGs and national events): therapists were asked to assess the themes generated from the FG, classifying them by whether they were ‘essential’, ‘desirable’ or ‘not used’ in their work. The classification was completed electronically, allowing responses to be collected and displayed anonymously. Discussion of the choices made by participants generated further insights into the purposes of participants’ interventions.
- Validation exercises (Round 3, national events): emerging challenges and issues with the themes were brought to the national events for discussion and exploration of consensus. Therapists were asked to electronically vote on challenges related to the names and definitions of the framework themes, as well as on issues of how the themes could be modelled. They were invited to construct a short vignette of a child with DS&LD for whom they had provided intervention, and later in the event, to describe what they did with the child (where relevant) in relation to each of the themes.

**Table 2:** Data outputs and analysis

Exercise	Therapists	Outputs	Analysis
Focus group	40	Transcripts of nine focus group discussions	Thematic analysis
Concept mapping	64	24 diagrams	Content analysis
Teach‐back tasks	37	Written descriptions of themes; of those contributing 26 SLTs provided descriptions of all 10 themes	Constant comparative analysis, deviant case analysis
Sorting task	64	Votes on whether themes were essential, desirable or not used	Descriptive statistics
Validation	62	Written description of therapy for a child in relation to themes	Consistency with themes, constant comparative analysis, deviant case analysis

Discussions that occurred during the concept mapping, teach‐back, sorting and validation exercises were captured in field notes, in participant notes and in researcher debriefing at the end of each event. Selected data from the data‐collection activities are presented in this paper; however, additional description of the data‐collection processes, examples of data and comments on the analysis are provided in the appendix.

### Analysis

The first data set, generated from the FGs, was analysed using thematic analysis, following the six stages set out by Braun and Clarke (2006), to explore the aims of therapy. Data collected in subsequent phases were analysed using techniques commonly seen in qualitative theory building methodologies such as Grounded Theory (Strauss and Corbin 1990); this included coding, thematic analysis, constant comparative analysis and deviant case analysis (Silverman 2006). The process was iterative with the analysis influencing subsequent phases of data collection and the gradual clarification of themes identified in the first round of data collection. Basic descriptive statistics (percentages) were used in the sorting tasks as a useful way to provide immediate feedback to participants about the level of consensus/agreement among them. Although not regularly used in qualitative research, they are cited in the present study to illustrate the level of consensus. Within the study itself, this information acted as a useful trigger to discussion amongst participants. The findings in this paper represent the concluding characterization of SLT practice with preschool children with DS&LD.

### Rigour

Several features and processes were built into the study design to ensure rigour. These all reflect the qualitative nature of the design and data‐collection processes and included the sampling processes, deviant case analysis, forms of respondent validation and triangulation and coding by multiple researchers. Further explanation is provided in the appendix.

## Results

### Participants

A total of 245 SLTs took part in the data‐collection events. The 40 therapists who attended the nine FGs had been qualified for an average of 14 years (range 2–43 years). Table 3 shows the number of participants involved in each data collection round and the overlap across rounds.

**Table 3:** Participant numbers for data‐collection stages

Data‐collection event	Therapists
Focus groups	40
Special‐interest groups	128
National events	90
Participants attending focus groups *and* special‐interest groups	10
Participants attending focus groups *and* national events	2
Participants attending special‐interest groups *and* national events	1
Total involved in data‐collection events	245

Thematic analysis of FGs initially generated 10 themes that sought to represent therapy aims for children with DS&LD. Where there were areas of contention, challenge and lack of consensus, further discussion took place in the third round national events. The validation exercises at these events resulted in changes to names for three themes and in two themes being merged, resulting in nine final themes. Changes were supported by therapists’ consensus in voting tasks. A total of 80% of therapists who completed the sorting exercise (*n* = 51) indicated that they thought that the original themes covered all aspects of their work with preschool children with DS&LD. Because of the anonymity of the sorting process, it was not possible to identify definitive reasons from any individual. However, if participants understood the task as related to their most typical workload, then it is easy to understand how some participants’ work might exclude particular components most of the time. In relation to the remaining 20%, one possibility was that the caseloads of some therapists may be composed mostly of children at the top end of the preschool age range and they may have focused on speech sound disorders. This was the only explanation offered during the discussions, but it was also evidenced by data from the validation tasks (see the appendix, tables A3 and A4). All therapists agreed that seven of the 10 themes were essential or desirable to their work. In the teach‐back exercises, the majority of participants provided explanations that were consistent (i.e., either the same or expanded) with the given definition. Examples of the range of responses is given in the appendix.

### Characterizing SLT

The final nine themes that represent therapy aims for preschool children with DS&LD can be conceptualized under three broad categories:

- Addressing the child's areas of impairment and skills.
- Achieving functionally meaningful skills and carryover.
- Supporting adults to provide a supportive communication environment.

These categories, and the final themes within them, are now described. Illustrative quotations are provided from FG data. Descriptions and summaries of themes reflect the findings from across the data‐collection activities. The sorting task data (table 4) are presented alongside the themes to provide evidence of the level of consensus in terms of the importance of each theme to therapy for children with DS&LD.

**Table 4:** Speech and language therapist (SLT) rating of themes (Turning point task)

Theme (*n* = 64)	Essential, *n* (%)	Desirable, *n* (%)	Not used, *n* (%)
Foundation skills	57 (88.9)	7 (11.1)	0
Comprehension	57 (89.1)	6 (9.4)	1 (1.5)
Expressive language	53 (82.8)	11 (17.2)	0
Speech/articulation	36 (56.3)	27 (42.2)	1 (1.5)
Sound awareness	40 (62.5)	24 (37.5)	0
Self‐monitoring	26 (40.6)	32 (50.0)	6 (9.4)
Generalization	56 (87.5)	8 (12.5)	0
Functional Communication	55 (85.9)	9 (14.1)	0
Adult understanding and empowerment	59 (92.2)	5 (7.8)	0
Adult–child interaction	59 (92.2)	5 (7.8)	0

### Addressing the child's areas of impairment and skills

When describing work with preschool children with DS&LD therapists often focused on target area(s) of a child's impairment and activities and strategies that they used to address it. Areas of impairment were discussed in broad terms and the following four themes were identified: foundation skills, comprehension, expressive language and speech. Foundation skills were deemed essential to children's speech and language development. If these were in place, therapists might progress to a range of direct work on comprehension, expressive language and/or speech. The exact goals were driven by the assessment process which determined the child's level in each of the four broad areas.

#### Foundation skills

Therapists described work to improve a range of skills that might be considered as foundations for speech and language development. The majority of participants (89%; table 4) reported that work on foundation skills was essential to therapy. This included working on a child's cognition and behaviour, in order for other learning to take place. Therapists reported activities and strategies that focused on promoting a child's turn taking, play, attention, selective attention and listening, nonverbal and social interaction skills:

Work around the kind of listening skills so their attention and listening erm their eye contact, their anticipation, so it's looking very much at those early pre‐verbal skills and assessing those at that initial appointment and determining whether or not we feel they're at a level in order to access what we're offering. (SLT_098)

#### Comprehension

As with foundation skills, 89% (table 4) of participants reported that work on comprehension was essential to their work. Therapists described activities and strategies that aim to improve the children's understanding of language. It was suggested that interventions in this area overlap with work on expressive language, particularly for vocabulary development. The comprehension tasks described predominantly focused on children following structured, play based, directions and varying the amount and variety of words that carry meaning in a sentence (information carrying words) within these tasks.

A bed, a chair, a table or umm fridge/sink and some people and ask the child to do some actions with them, make the man jump on the table, make the boy hide under the table, so on one hand. … I'm checking their ability to follow that kind of instruction and we're also feeding in the verb because I find an awful lot of my children know nouns, but don't seem to have come very far with verbs and build up language in that way, and using very short sentences. (SLT_061)

#### Expressive language

Therapists described work that aims to improve children's expressive vocabulary and the structure and the length of utterances. This aspect of work was reported as essential to practice by 83% of participants. Targets include the production of single words and new vocabulary (especially verbs), putting words together, and some grammar and morphology. Typically, language activities were encouraged in play‐based settings in the children's everyday lives. This included specific play activities (e.g., playing with bubbles) to model target words, or using objects to practise naming and verbs. Therapists also described more general situational learning, such as encouraging the nursery to have vocabulary themed weeks.

Parents have certainly come back and said ‘they're going up, up, up up’ because I have a ladder, which is a great thing for the little man that goes up up up, … so it's taking a framework of a situation that you can hold the children's attention, give them something that they are interested in doing and then make the language the important bit of it. (SLT_054)

#### Speech

Therapists reported activities and strategies designed to increase the accuracy of speech production or articulation. This included work on speech production such as drill type activities or sounds in isolation, working in a hierarchy of sound production (e.g., from single consonants, to consonant–vowel production), cued articulation and blending. Following FGs, sound awareness was initially identified as an additional theme to speech. However, in subsequent KE tasks, and in a final vote at national events, it was found that the majority of participants considered sound awareness tasks key to work that supported speech development and the two themes were merged. Thus, speech activities include activities focusing on phonological awareness, auditory bombardment, syllable counting, minimal pairs and discrimination of sounds such as front and back sounds. The majority of participants (56.3%) reported work on speech is essential, and 62.5% reported work on sound awareness was essential; most of the remaining participants classified this work as desirable.

I would then target some … either words with them in or I might work with some sounds just at that single sound level although … with children that young it would still be a lot of auditory bombardment work primarily, especially if they can't do it at all. If they're able to do it in isolation but not in words then I would … practice it both in listening and in production at this sort of CV VC levels first. (SLT_004)

### Achieving functionally meaningful skills and carryover

Therapists described a range of strategies intended to help the child communicate meaningfully outside the therapy environment. These included providing the child with skills to improve awareness of their difficulties (self‐monitoring) as well as structuring activities to encourage SLT targets to develop outside therapeutic settings (generalization). Finally, therapists encouraged alternative methods to facilitate communication and understanding of information (functional communication) such as the use of manual signing and pictographic symbols.

#### Self‐monitoring

Therapists reported activities designed to help the child develop awareness of their own speech and language difficulties and how they might be able to overcome them. Most commonly participants referred to the self‐monitoring in relation to speech output, although it might focus on expressive language skills. Therapists described specific strategies including using tokens to provide feedback, as well as more general activities, for example, discrimination, encouraging children to reflect on their speech and self‐correct or repair a communication event. Only 40% of participants in the voting activity perceived this to be an essential part of their work, and 9% reported that they did not work on this.

I would only do the kind of tongue twisters self‐monitoring stages if they were really able in that age group, but I tend to use kind of tokens ‘cos that's the self‐monitoring bit … is the bit that I always find is quite a big lip to get over. (SLT_002)

#### Generalization

The majority of therapists (87%) reported that generalization of therapy gains to non‐clinical situations and environments was essential to their work. They referred to the importance of parents and other adults working with the child to use activities and strategies in different contexts, to encourage generalization. However, they rarely specified activities used to achieve it.

reinforcement and carry over at home, to get, to get, otherwise they're just not going to move forward so it's getting that carry over isn't it. (SLT_103)

#### Functional communication

Therapists described strategies and activities that help the child's involvement and participation in life situations, this work has been termed ‘functional communication’. Work in this area was reported as essential by 86% of therapists, and a further 14% reported it was desirable. Interventions might include using signing or symbols to help a child communicate, the use of visual approaches to support comprehension (e.g., ‘now and next’ boards), ensuring children's social participation in a small language group as well as extending children's range of play to encourage them to join in with other children. Other therapists described work on emotion and reasoning in social situations.

To have the visual support to help them to understand the words and to link that umm, or for expressive language, to enable them to actually participate and to make a choice, or to make their needs known because often, especially in nursery … the child is not getting their needs met or not (being) able to communicate. (SLT_017)

### Supporting adults to provide a supportive communication environment

The third theme that was identified concerned the role of adults who work with the child. In the FGs, therapists talked about how they work with parents. Further exploration in KE sessions established that this kind of work also included all adults who spent time with the child. Therapists reported a common purpose in ensuring that any significant adult, who spent considerable time with the child, understood both the nature of their impairment and the role that they play in supporting them. Many therapists talked specifically about the kinds of changes that adults need to make in their interactions in order to support the child's speech and language development.

#### Adult understanding and empowerment

Therapists described work to help parents and other significant adults to understand the nature of their child's speech and language difficulty, what helps to improve it and why. An important aspect of this was described as developing their understanding of their role as a ‘major tool of change’ (SLT_099). Therapists rarely reported specific activities or tools in this area, but talked about providing explanations and information either in clinical or training sessions, including training for early years practitioners. This work appeared to be a common feature of everyday practice with 92% of participants reporting it as essential and the other 8% indicating that it was desirable.

It's about changing a parent's perception of what (therapy group) is about isn't it and helping the parents to take on board the fact that they have some input in to changing or supporting, developing this child's language. (SLT_106)

#### Adult–child interaction

Therapists emphasized working on adult– or parent–child interaction, to encourage speech and language development. They reported working on interaction strategies including sitting and playing with child, following the child's lead, commenting on the child's activities, or reducing questions to the child. Therapists referred to the importance of improving the ‘communication environment’ (SLT_095). They talked of providing advice and recommendations for specific activities to individual carers as well as within groups. Some therapists described active therapy, focusing on observing or recording a carer's interaction skills and working to help the carer develop specific interaction strategies. Many therapists reported modelling approaches for carers, encouraging carers to implement these approaches themselves, with further observation or follow up sessions to see if the strategies were being used. As with ‘adult understanding and empowerment’, 92% of participants indicated that this work is essential.

We model don't we, how to do it … sometimes again I overtly draw attention to that and say at the end of the session ‘did you notice that I said, ready, steady, go?’ or ‘bubble’ ‘did you notice that’ and that I said it lots of times. By the end of the session he was copying me. (SLT_099)

### How the themes fit together

**Figure 1:**
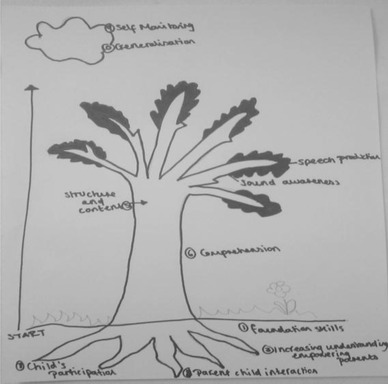
Hierarchical tree model.

**Figure 2:**
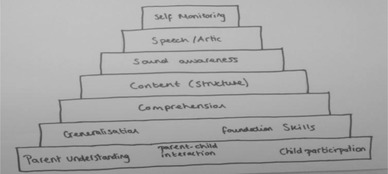
Pyramid model.

**Figure 3:**
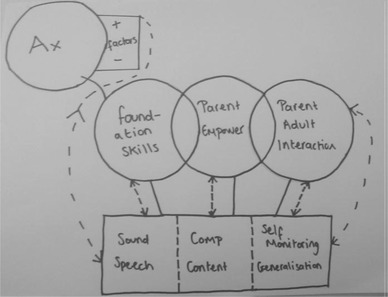
Balloon model.

**Figure 4:**
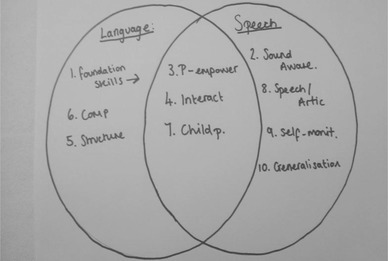
Venn diagram modular model.

The concept mapping tasks asked participants to model how the areas of practice related to each other, resulting in 24 representations. The models produced can be divided into two groups, those that were hierarchical and those that were modular. For the two types of model there were, however, some overlaps in therapists’ descriptions. Figures 1 and 2 are hierarchical models which include laddered schemas or flow charts suggesting ordered themes that were broadly developmental or progressive. These hierarchical models suggest that some areas of practice are regarded as more fundamental than others. Figures 3 and 4 are modular representations of practice, taking the form of boxes, balloons and Venn diagrams, showing relationships between the modules.

At the validation exercises at the SIGs, participants (*n* = 90) were asked to indicate their beliefs about these conceptual relationships. The majority (76%) agreed that their practice was ordered although opinion was divided on whether the modular or hierarchical model (49–52%, respectively) was the best fit.

### ‘It depends’—the individualization of intervention

The framework presented here provides a broad overview of therapy for DS&LD; it does not describe the process of how SLTs select their interventions.

The notion of individualization underpinned therapists’ descriptions of their practice. Their descriptions of therapy across all events stressed that therapy was not formulaic, but adjusted in response to the context, needs and responses of the child. In the concept‐mapping task, the relationship between the assessment process and the selection of intervention components is illustrated through the model in Figure 3. Additionally, data captured in the vignettes (see the appendix) illustrated the link between the presenting symptoms of the child and the use of particular components.

Irrespective of the KE task, participants constantly provided caveats regarding the intervention approaches they described. Although specifically asked what would make them vary their intervention, the phrase ‘it depends’ or variants thereof was pervasive throughout therapists’ responses. The ‘depend’ word was frequently accompanied by an ‘if *x* then *y*’ rule, where a certain context would give rise to a particular action. The factors that were described as influential included: the child's developmental level; their needs and progress; the parents’ engagement with therapy; the target of intervention; the practicalities and resources available and aspects of the therapist's role. When discussing these caveats, therapists used words such as ‘personalize’, ‘individualize’ and ‘flexibility’.

I do a phonological awareness … 3 or 4 weeks depending on what the child understands about sounds. If it is just one or two sounds, if they can get the sound on their own (then) I tend to send them away and they get on their own. (SLT_100)

That's what they pay us for is that we personalise then within that group situation. So … we can be flexible around that depending on what the parent's needs are. … I would individually speak to each of the parents … we would then focus them individually on what we would like them to do with their child so although it's group therapy it's. (SLT_095)

it's individual. (several SLTs)

It is a very individualised and it can be absolutely exhausting can't it [laughter]. (SLT_096)

## Discussion

There are ongoing challenges to the implementation of EBP practice in SLT. Although therapists are encouraged and supported to identify and appraise research, there is little in the literature about the nature of practice, including how therapists conceptualize therapy and integrate evidence into their practice. This work makes current practice explicit and provides a framework that describes how therapists structure their practice. This in turn facilitates the matching of relevant evidence from the literature to practice.

This study took a predominantly qualitative approach using KE techniques to explore SLTs’ practice with preschool children with DS&LD. Therapy for preschool children with DS&LD was conceptualized under three categories; addressing the child's impairment, functionally meaningful skills and supporting adults to provide a supportive communication environment. The process of selecting interventions was portrayed by therapists as an individualized process that is dependent upon a wide range of factors including the profile of the child, the parent, resources available and their role in a particular service.

### Addressing the child's impairment

A traditional or medical model holds expectation that therapists are aiming to improve underlying impairments and it is hardly surprising that they described work in this area. Therapists talked about core areas of impairment, such as foundation skills, comprehension, expressive language and speech. Within the literature, there are a myriad of complex underlying theoretical models related to these areas. For example, in the realms of speech, a number of models have been described and used in the literature to suggest interventions (Holm and Crosbie 2006). However, no pre‐existing theoretical model emerged in the discussions with and between therapists. This may be because therapists simplify and use their own version of theory, making complex ideas relevant and appropriate to their own practice context, as suggested by Boschuizen and Schmidt (2000). Alternatively, it may reflect the range of complex theories in the different areas of impairment, with evidence so far failing to support any one particular approach over any another (e.g., Wren *et al*. 2018).

### Functionally meaningful

Since language is the major tool of socialization and learning, it is crucial that any speech and language work considers the benefits to the child's development and socialization. Indeed, nearly 86% of therapists who completed the sorting task, classified this work as ‘essential’.

The importance of working on functionally meaningful aspects of language has been described elsewhere; two studies exploring interventions for school‐age children with receptive language impairment (Law *et al*. 2008, Morgan 2013) have indicated that therapists are more concerned with the impact of impairment rather than the impairment per se. Furthermore, studies that have investigated the outcomes valued by children and their parents have found repeatedly that the functional outcomes are the ones that are valued most (Roulstone *et al*. 2012a, Beresford and Sloper 2003). Despite the importance ascribed by therapists and parents, this aspect of intervention has not taken prominence in the research literature. A systematic review of intervention studies for preschool children with DS&LD (Roulstone *et al*. 2015) included 58 studies, of which only five included activities that targeted functional communication, with the primary focus of these studies tending to be expressive language, for example, grammar/syntax or vocabulary production, rather than functional communication.

### Supporting adults

The emphasis on supporting adults to change their interactions in order to support a child's language development is not a new concept (Pickstone *et al*. 2009). However, there is rarely a focus on the actual process of training or supporting the adults. The systematic review by Roulstone *et al*. (2015) found that only one of the 58 studies measured parental understanding of the intervention. Klatte and Roulstone (2016) found that therapists considered that parents’ engagement, parents’ understanding and their ability to reflect on their own interactions were crucial components in parent–child interaction therapies, but these were rarely explicit in the related literature. In other healthcare fields such as diabetes (Gatzoyia *et al*. 2006) and autism (Lawson *et al*. 2004), evidence indicates that parents’ understanding and beliefs about their child's impairment affects their engagement with services, adherence to treatment and their general well‐being (Horne *et al*. 2013), demonstrating the potential power of approaches that target parent understanding.

In the present study therapists made clear that parent understanding and empowerment were important features of practice; however, the details of how their work ensured parents’ development in these areas were underspecified. Davies *et al*. (2016), found that parents see their role as advocates for their children. For some parents this shifted following SLT input, from perceiving themselves as advocates to implementers and adopting more active roles as interveners in their child's speech and language development. However, further work is needed to understand the nature of interventions that support parents’ behaviour change.

### How the themes fit together

The models of themes are varied, reflecting the complexity of the relationships of therapy targets to one another. Where models were modular, a number included feedback loops (e.g., model 3), and others overlapping themes/modules (e.g., models 3 and 4), which provide further evidence of the complexity in modelling and classifying therapy themes.

The prevalence of hierarchical models suggests some areas of practice are regarded as more fundamental than others. Indeed, the majority of participants agreed that there was some form of order to the models (76%), and most placed the themes adult understanding and empowerment, adult–child interaction, foundation skills and child participation, at the bottom of the model. Comprehension was frequently toward the bottom, but sometimes placed above these themes. These ‘core’ themes had the highest rates of being categorized as ‘essential’ to SLT in other data‐collection tasks, with more than 85% of participants putting them in this category.

The use of hierarchical models in SLT has recently been a topic of debate both on Twitter and in the *RCSLT Bulletin* (Morgan and Dipper 2018). It has been suggested that a particularly prevalent hierarchical model in SLT practice ‘The Communication Pyramid’ (e.g., The Communication Trust 2011), has no obvious academic evidence to support it. Morgan and Dipper (2018), argue that, the pyramid suggests that skills develop consecutively whereas evidence demonstrates, for example, that attention and listening, and speech develop alongside each other, not first and last, as indicated in such models. In the present study, SLTs’ hierarchical models show some order to therapy and that some themes are a priority for intervention before others. This is arguably different from indicating that areas for therapy are independent of each other, or that there is strict order to the themes. Indeed, discussion indicated therapists do not believe therapy to be this simplistic and some drew models with overlapping themes. In their practice, therapists attempt to translate highly complex, competing and interacting theories of speech and language development into something concrete that can be operationalized for practice and that can be readily explained to others. It could be that this regular practice reduces therapists’ fluency or facility or confidence to explain the more subtle nuance and complexity of their practice.

Most participants emphasized the importance of the themes adult understanding and empowerment, adult–child interaction, foundation skills and child participation. This is perhaps unsurprising since there is less direct therapy in the early years and arguably, this period offers the greatest chance for parents/adults to make a real difference (Roulstone *et al*. 2012b). Preschool children are also less likely to have some of the core foundation skills (attention, turn taking) and without these, it is hard for children to engage in specific speech and language activities in any meaningful way.

The high value therapists place on functional communication is demonstrated by the fact that more categorized this theme as ‘essential’ to their work for this age group, than work on expressive language. At the FGs participants spoke of providing ‘means, reasons and opportunities’ for children to communicate, and it is likely that it is this, participatory aspect of the theme that makes it particularly core to communication development.

### Individualization

It was noted at the start of this paper that a striking feature of SLT practice is its variation. Indeed, it is well established that there are a wide range of interventions for children with DS&LD, for example Roulstone *et al*. (2012a) identified over 150 interventions used with this client group. In a study exploring practice for children with phonological difficulties, Joffe and Pring (2008) refer to the ‘eclectic’ approach of clinicians. Although we know some variation in interventions is determined by the age of the child, the diagnostic category and the setting (Law *et al*. 2008, Roulstone *et al*. 2012a), there appears to be little written about variation being due to therapists’ individualization of practice. While the concept of individualization is likely to chime with many clinicians, it is not well described in the literature.

On the one hand, individualization is an important feature of EBP where a therapist uses their clinical expertise to appropriately adapt interventions to the child and context. On the other, it creates a conflict for the creation of evidence, particularly in research that seeks consistency, in order to reduce bias. It also causes conflict with models of practice that emphasize broad care pathways that children are assigned to based on broad patterns of presentation.

The challenge for researchers is to incorporate individualization of therapy described in this study into robust research evaluation designs. Some researchers (e.g., Adams *et al*. 2006, Boyle *et al*. 2007) have developed manualized SLT programmes, which incorporate a range of specific interventions that can be individually designed to mirror a child's profile. The framework presented in the current paper provides a useful and flexible overview of the features of therapy. By building on this work and specifying the intervention activities and strategies within therapy themes, as well the evidence associated with these, it will be possible to create a similar manualized approach which can be suitably individualized, yet holds consistency within it.

### Applying the framework

The framework provides a high‐level structure of what therapists consider to be the critical tenets of practice for preschool children with DS&LD. It has been designed to reflect the way that therapists talk to others about their subject, in an accessible format. This overview cannot contain all the competing theories and models of speech and language development that exist. However, therapists are using their own adaptations of these theories and integrating them for individual children who present with different profiles of impairment, often not isolated to one area. A strong feature of the framework is that it allows for this flexibility and adaptations of therapy described by therapists. Further, the process of validation and consensus used in the present research indicates that, at a high level, the framework represents all aspects of their work. What the framework does not yet do, is establish the details of therapy (dosage, method, delivery and critical components), as well as what is an acceptable range of variation in practice. It is clear that SLT practice is complex; therefore, the way in which we describe our interventions and the frameworks that we use to illustrate practice, are ongoing challenges.

Roulstone *et al*. (2015), have used the framework to investigate the intervention evidence for each theme; their work highlighted areas of practice that are important to therapists, which are currently under‐investigated in the literature. In particular, there is a lack of research focusing on functionally meaningful aspects of speech and language learning and those focusing on parent understanding. Work needs to be done to plug this evidence gap.

### Strengths and limitations of the research

The broad and iterative nature of the sampling process provides some confidence that the findings reflect the range of views across the profession working with this group of children, although they cannot be regarded as representative. It is noteworthy, however, that there was a good level of agreement about the high level components of practice. When working at the level of more granular definitions and details of interventions, it is perhaps likely that more variation will emerge.

## Conclusions

The framework of therapy presented in this paper provides a characterization of SLT for preschool children with DS&LD. It begins to bridge the gap between practice, theories of therapy and evidence of interventions. While further work is needed to provide details of interventions and models that sit within the framework, it takes an important first step in highlighting SLTs’ priorities for preschool children with DS&LD.

The present study used KE techniques designed to facilitate the explicit sharing of implicit or tacit knowledge (Rycroft‐Malone *et al*. 2004: 83). However, even with these techniques using explicit, clearly defined terms, activities and strategies, it is difficult for therapists to clearly articulate what is inherently tacit knowledge. This may in part reflect what are by nature complex interventions.

The framework, which is validated by SLTs, indicates important directions for future research. This includes intervention studies that focus on functional aspects of communication, as well as those involving supporting adults to improve the communication environment. The research highlights the complex nature of interventions for preschool children with DS&LD and the importance therapists attribute to tailoring therapy to individual needs, with ‘it depends’ being a by‐line of the study.

## Appendix A Additional material on methods

‘Child Talk’ was a large multistage mixed method study. The full report is available online, published by the NIHR as part of the Journal ‘Programme Grants for Applied Research’ (Roulstone *et al*. 2015) (https://www.journalslibrary.nihr.ac.uk/programmes/pgfar/RP-PG-0109-10073/#/). This appendix focuses on those particular data‐collection activities and analyses of relevance to topics of this paper, that is, the characterization of speech and language therapy (SLT) with preschool children with primary speech and language impairment (now referred to as development speech and language disorders). This appendix provides further details of the sampling process, data collection, additional examples of data and comments on analysis.

### Purposive sampling of six NHS sites

A literature search by the Child Talk team identified factors that were reported to impact on SLT service provision. Six factors were identified: ethnic minority populations, socioeconomic status, urban/suburban/rural, transience of population, bilingual populations and Early Years Foundation Stage (EYFS) scores. Data from nationally available statistics for these factors were mapped onto geographical areas in England to identify potential SLT services. The individual sites within each of these groups was assigned a score from 1 to 6, with 1 representing ‘low’, for example, a low level of ethnic diversity, to 6, representing ‘high’, for example, a high percentage of pupils scoring well on EYFS. The data were then scrutinized and sites were identified that provided a range of scores on each variable. From this mapping exercise six case study sites were chosen, which provided a spread, across the categories, to invite to become case study sites. Potential sites were approached by the research team through discussions with the SLT service lead. If a service was not in a position to become a site, the data were re‐examined to find a new site with a similar spread of scores. It took three iterations to recruit six. The final six sites all presented with a different set of scores on the six factors. Thus, the sample was purposive and provides services with a range of factors that are likely to impinge on how they organize their services and deliver interventions. By this means, the project aimed to sample the widest range of experiences from across England, although of course it cannot be guaranteed that we have sampled every possible variation. A full account of this sampling process is available in the project report (Roulstone *et al*. 2015: 12).

### Knowledge elicitation data‐collection techniques

Knowledge elicitation (KE) is a term found in the field of artificial intelligence (AI) and knowledge engineering. In these fields, the software engineers are attempting to capture the knowledge of professionals and organizations and to replicate or improve on expert decisions through computerization of those decisions. Professional knowledge has a number of characteristics that challenge the researcher in terms of selection of data‐collection processes. It is complex and highly contextualized often with many interacting components, with specialized discourses; most challenging of all, much of it is tacit and not readily communicated. This is because, as professionals develop their expertise, their knowledge becomes embedded or ‘encapsulated’ (Rikers *et al*. 2000) in the context of their practice; specialist knowledge (such as biomedical knowledge) becomes linked to various clinical problems and presentations. The process of eliciting such knowledge requires data‐collection techniques that require the respondent to reflect in detail on their clinical context and to make explicit the knowledge that they access in certain clinical contexts. KE techniques have been used in a wide range of professional contexts such as medical informatics (Reese *et al*. 2018), the exploration of how experts assess risk in the process of identifying an emergency (Whitmer *et al*. 2016), and in a quality assurance project to develop a business strategy (Yip and Rongbin 2017).

In this project, focus groups were used to generate an initial data set that allowed the generation of a conceptual framework, in our case a set of themes. Subsequent data collection provided feedback to participants about those themes and asked them to reflect on how far the framework mapped onto their practice. KE exercises therefore focused on providing situations to stimulate reflection on the knowledge used in certain contexts.

Further details follow of the particular KE activities that were used in this project.

**Table A1:** Abbreviated topic guide for focus groups

Setting ground rules
Define significant terminology.
Discuss and add to a provided list of SLT‐led interventions
Discuss and add to a list of SLT‐led intervention targets
Discuss the *critical components* of SLT‐led intervention for pre‐school children with PSLI.
Discuss the *factors* and *influences* that might result in the modification of intervention activities and strategies.
Discuss *how* intervention might be modified
Discuss the *factors* and *influences* that might result in the modification of intervention targets.
Discuss *how* targets might be modified

### Round 1 focus groups—abbreviated topic guide (table A1)

A full version is available in the published report (Roulstone *et al*. 2015).

### Concept mapping data collection and analysis

Concept maps provide a useful way both to elicit and display the knowledge of a domain. They have been used to explore and compare the knowledge of novice and experts and to achieve consensus between experts (Crandall *et al*. 2006). The concept map is a diagram that represents the knowledge of an individual or a domain, making relationships between concepts explicit. Typically, a researcher would co‐construct a concept map with the participant, the researcher asking questions and providing suggestions and alternatives in order to clarify concepts. In the Child Talk study, we used a simplified version of the process, asking participant therapists to discuss with each other and capture how they felt the themes from the focus group analysis related to each other. They were encouraged to capture their discussions in a diagram and then to present these to the rest of the group. Examples of these are presented in figures 1, 2, 3, 4 in the main text. The diagrams were all displayed together and were then analysed jointly by three of the research team (SR, GP, JM). Following familiarization, the team conducted a process of constant comparison, looking for similarities, differences and patterns until consensus emerged within the team about the ways that participants were combining and organizing the themes.

### Teach‐back data collection and analysis

‘Teach‐back’ is a common technique in education and health contexts for checking someone's understanding of information received. For example, in a patient consultation, a doctor may use teach‐back techniques to check that a patient has understood directions for taking medicine. The aim is not to test the patient's knowledge but to check that the doctor has explained clearly enough. Therefore, a patient may be asked: ‘So that I can check that I have explained it clearly for you, could you explain to me how you will take your medicines after today.’ The technique has been used in a KE context to check that the researcher has understood the professional's explanation of a process or decision (Johnson and Johnson 1987).

In the Child Talk study, we adapted the technique as a means of checking the researcher's thematic analysis of focus group data and generating new data around each theme. Participant therapists were provided with summaries of each theme. They were then asked to explain that theme as if they were explaining it to a parent, thus requiring the participant to show their own understanding of that theme. In a process of constant comparison, the participants’ explanations were then compared with each other within and across themes and with the researchers’ descriptions of the themes. For the purposes of this paper, this level of analysis was used to validate the thematic analysis, checking that the themes were non‐overlapping or for varying interpretations. Three examples of the data are given below (table A2) with respect to the theme ‘comprehension’. As can be seen from the three examples, participants interpreted the task and the theme differently. In some cases, the content was similar but in more or less detail (example 1). In others, participants chose to focus on different aspects of the theme, for example, the use of visual cues (example 2) or the notion of impact on functionality (example 3).

**Table A2:** Participant explanations to parents of ‘comprehension’

Example 1: To help your child understand what people say to them. To help them understand language and not just rely on the clues in the situation.
Example 2: Language is very complicated for young children to learn and understand. They often use cues from the situation, e.g., if you point to something as you ask them to bring it to you they can do what you say, but if you just say ‘pass me the ball’ they may not understand the word ‘ball’ yet. Or he may put his shoes on when you are going out because he knows the routine, not because you say put your shoes on. We need to help him learn to decode what the words mean and that will be really helpful for him when he starts nursery ….
Example 3: A child needs to understand what is happening at home and at preschool, in order to join in with games and follow instructions. We need to make sure that his understanding of language is good before we can start working on talking skills

**Table A3:** Boy aged 4 years 0 months presenting with severe attention and listening difficulties, receptive and expressive language impairment, weak social skills (secondary), receptive (moderate), expressive (severe), single‐word level

Foundation skills	Intensive attention‐building programme; direct SLT‐led (fortnightly) and three times by teaching assistant trained up
Comprehension	Keyword‐level work and concept development
Expression	Vocabulary‐building via talking box work. High focus on high frequency nouns and verbs
Speech	Advised school staff to follow phase I letters and sounds
Self‐monitoring	Awareness‐raising when he got over‐excited about what he needed to do to be calm
Generalization	Solution‐focused conversation with parents and staff on what we could see him do when he had generalized his skills and translated into measurable outcomes
Participation	Followed child's interests; saw in a group of three; lots of high interest, fun activities
Parent–child interaction (PCI)	PCI strategies, whole school training to promote adult–child interaction (ACI)
Parent understanding	Parent consultation termly or on request. Solution focused model

**Table A4:** Girl aged 3 years 4 months presenting with disordered speech which was impacting on grammar in expressive language

Foundation skills	Basic listening games, musical instruments, LDA environmental sounds; range of sound picture matching games
Comprehension	Assessment using CELF‐Preschool and reports of home and preschool
Expression	Some games to reinforce use of /s/, /ed/ as grammatical markers
Speech	Auditory discrimination/minimal pairs and production work based on psycholinguistic framework
Self‐monitoring	Lots of games/recording story telling—informal and support for family to help child reflect on self and self‐monitor speech
Generalization	New school so will be setting targets for class teacher, school staff, home to report on child's intelligibility/number of times they need to ask child to repeat herself clearly
Participation	Simply monitored this. Child remained participative despite speech disorder
Parent–child interaction (PCI)	Diagnosis and advice to teachers and family members, that is, supporting a mainstream child's confidence to chat/communicate
Parent understanding	Sessions with parents observing, tasks used to identify where within psycho‐linguistic framework activities needed to target

**Table A5:** Boy aged 2 years 8 months presenting with delayed language development, approximately three words (expression) at initial assessment; delayed play and social interaction

Foundation skills	Activities to support attention, play, early social skills
Comprehension	Programme provided to parent with early receptive vocabulary activities and advice on visual cues
Expression	
Speech	
Self‐monitoring	
Generalization	
Participation	Programme also contained advice on early social skills and shared attention
Parent–child interaction (PCI)	
Parent understanding	Parent attended three workshop sessions, explained early communication skills and importance of play; programme to provide parent with advice about daily special time

### Sorting task data collection and analysis

Asking knowledge domain experts to sort concepts in different ways is one of the more contrived and controlled ways of exploring and eliciting knowledge. The task may require experts to organize their knowledge in an unfamiliar way that exposes some of their more tacit knowledge. In Child Talk we used an electronic voting system to ask participants whether they considered each of the themes to be essential, desirable or not used in their work. The electronic version allowed the research team to capture the number of participants ‘sorting’ each theme whilst maintaining anonymity for participants, thus making it more likely that participants’ votes would be representative of their usual practice. The purpose of the task was to check the validity of the thematic analysis and to use instance of disagreement to explore a theme further. Thus, the process itself became an instance of deviant case analysis, whereby the discrepancy was used to challenge the coherence of the thematic analysis. The electronic system allowed participants to suggest and discuss reasons for discrepancies without disclosing their own voting decisions.

The results from the votes have been presented within the main text as part of the validation for the thematic analysis—showing that generally the thematic analysis has captured what participants consider to be the central components of their work with preschool children.

Instances of disagreement were used as a basis for discussions in further data collection and subsequently to update the definitions and characterization of themes. The main source of disagreement was the lack of clarity and understanding of the theme definitions, and what they encompassed, rather than the complete absence of important categories. Reflections by the team on the discussions led to a realignment and renaming of themes, particularly those causing disagreement during the discussions. This realignment was put to the national meetings and indications from those discussions and validation exercises suggested that the final themes were inclusive and comprehensive

### Child vignettes as a means of validation

Participants in the national events were provided with proformas of the thematic analysis and asked to use that framework to describe their work with a child from their caseload. They were asked to describe intervention activities and any assessments or measures they had used in connection with each theme. Examples of vignettes showing the ‘interventions’ listed for each theme are given below. As can been seen from these examples, not all cases used all themes. However, across all the cases completed, all the themes were used confirming that the themes had captured the totality of work with preschool children with DS&LD. The variability across vignettes also illustrates how the balance of work changes in relation to the specifics of the child's presenting symptoms which would be identified by assessment. This relationship between assessment and the selection of components was also seen in the concept mappi`ng process (e.g., see figure 3 in the main text which makes explicit the link between Assessment (‘Ax’) and investigation of relevant factors which then feed into the selection of components).

As with other validating data, a process of constant comparison and deviant case analysis was used to review the extant thematic analysis in the light of the new data collected via the vignettes. Few adjustments were needed to the themes at this stage; however, the analysis added to our understanding of how components are used and our descriptors of each.

### Rigour

The achievement of rigour must begin in the planning stages rather than merely used at the end of the study to evaluate a study (Cypress 2017). Typical parameters used to consider the rigour of a qualitative study have included trustworthiness, credibility, transferability, confirmability (Bryman 2008). Yardley (2000) suggested that rigour should cover sensitivity to context, commitment and rigour, transparency and coherence, impact and importance. Examples of how the design in this study planned for rigour are shown in the following processes:

- *Sampling*: purposive sampling was used to ensure that participants came from a range of practice contexts. Sampling of this kind is not aimed at producing a representative sample but makes it more likely that the experiences of different SLTs working with preschool children in England has been captured, thus increasing the possibility that the findings of the study are transferable.
- *Deviant case analysis*: the data‐collection techniques aimed to provide participants with contrasting ways of expressing their practice knowledge. The first data set from the focus groups was used to generate the basic analysis; subsequent data collection was used to test the hypotheses about practice that were captured within that basic analysis; deviant cases or negative instances that appeared to contradict that basic analysis required an adjustment of that basic analysis, thus validating and improving the credibility of the final analysis.
- *Respondent validation and triangulation*: the initial analysis of the data from the focus group was discussed with subsequent participants to examine its validity or credibility in terms of how well it captured their practice; the variety of data‐collection methods provided a form of triangulation to test whether the different ways of exploring practice generated similar or conflicting data.
- *Coding by multiple researchers*: the nature of qualitative research involves a level of subjectivity in that the researcher is required to interpret the meanings in the data. Coding by multiple researchers allows a challenge to the individual researcher and the ensuing analysis is arrived at through discussion and consensus thus guarding against the particular orientation (or bias) of any one member of the research team. The initial analysis was completed by the first author LM, SR viewed transcriptions independently and identified themes. These were then compared and finalized in discussion. Subsequent data were considered by three authors (JM, GP and SR) arriving at a consensus position through discussion.
